# Clinical characteristics and pregnancy outcomes in polycystic ovary syndrome complicated by obstructive sleep apnea: a correlative study

**DOI:** 10.3389/fmed.2026.1714294

**Published:** 2026-04-15

**Authors:** Jiena Li, Ya Liu, Xiangbin Zheng, Dan Cao, Yuanyuan Wu, Xiaofan Huang, Bo Chen

**Affiliations:** 1 Department of Obstetrics and Gynecology, Center for Reproductive Medicine, Heze Hospital Affiliated of Shandong First Medical University, Heze Municipal Hospital, Heze, Shandong, China; 2 Shantou Key Laboratory of Basic and Translational Research of Malignant Tumor, Clinical Research Center, Shantou Central Hospital, Shantou, Guangdong, China; 3 Department of Laboratory Medicine, Heze Hospital Affiliated of Shandong First Medical University, Heze Municipal Hospital, Heze, Shandong, China

**Keywords:** AMH, insulin resistance, obstructive sleep apnea, polycystic ovary syndrome, pregnancy outcomes

## Abstract

**Background:**

Polycystic ovary syndrome (PCOS) is associated with a high prevalence of obstructive sleep apnea (OSA); however, its impact on the clinical manifestations of PCOS remains unclear.

**Objective:**

This study aimed to investigate the clinical characteristics, reproductive endocrine profiles, and glucose–lipid metabolic features in women with PCOS complicated by OSA and to evaluate their potential implications for pregnancy outcomes.

**Methods:**

A total of 294 infertile women with PCOS treated at Heze Municipal Hospital between February 2023 and December 2024 were enrolled after excluding ineligible participants. Clinical characteristics and reproductive endocrine and metabolic parameters were collected, and factors associated with OSA in women with PCOS were analyzed using the univariate analysis and the logistic regression analysis. Additionally, 101 women with PCOS and OSA were randomly assigned to either a lifestyle intervention control group or a lifestyle intervention and continuous positive airway pressure (CPAP) group, and changes in hormonal and metabolic parameters as well as pregnancy outcomes were evaluated.

**Results:**

The prevalence of OSA in women with PCOS was 34.4%, of whom 10.9% had moderate to severe OSA. Higher body mass index, hyperinsulinemia, elevated triglycerides (TGs), and reduced anti-Müllerian hormone (AMH) levels were independently associated with an increased risk of OSA in women with PCOS. Compared with the control group, the CPAP treatment group exhibited significant reductions in serum testosterone (T), insulin (INS), and triglyceride (TG) levels, along with a significantly higher clinical pregnancy rate (*p* < 0.05).

**Conclusion:**

Infertile women with PCOS and OSA exhibit significant disturbances in reproductive endocrine and glucose–lipid metabolism. CPAP therapy may improve these metabolic abnormalities, and when combined with ovulation induction and assisted reproduction, it may lead to better pregnancy outcomes, highlighting the potential benefit of early screening and intervention for OSA in women with PCOS.

## 1 Introduction

Polycystic ovary syndrome (PCOS) is the most common endocrine disorder, affecting up to 13% of women of reproductive age worldwide (1). The pathophysiological features of PCOS in women, such as insulin (INS) resistance, hyperandrogenism, and chronic low-grade inflammation, may increase the risk of obstructive sleep apnea (OSA) (2). OSA is defined as an apnea–hypopnea index (AHI) ≥5 events/h in the presence of characteristic clinical manifestations, including excessive daytime sleepiness and/or cardiometabolic comorbidities, such as hypertension (3). In a large-sample study conducted in Europe, Kumarendran et al. (4) enrolled women with PCOS as the study group and women without PCOS as the control group, indicating that the risk of developing OSA was significantly higher in the PCOS group than in the control group. A majority of the studies have shown that the prevalence of OSA is significantly higher in patients with PCOS than in the general population (5). Although the mechanism of its occurrence is not fully understood, patients with both PCOS and OSA share key clinical characteristics of PCOS, including obesity, sex hormone levels, and indicators of glucose and lipid metabolism, with obesity probably being the most important factor (6). Women with PCOS and OSA exhibit more severe sleep disturbances and higher apnea–hypopnea indices than those without OSA.

PCOS contributes to the development of OSA through multiple underlying mechanisms, including insulin resistance, hyperandrogenemia, and chronic low-grade inflammation (7). In patients with OSA, repeated apneic episodes and recurrent cycles of hypoxia and reoxygenation trigger the release of multiple inflammatory mediators, while inflammation and oxidative stress may further exacerbate the pathophysiological processes of PCOS (8). Additionally, hyperandrogenemia in PCOS increases susceptibility to OSA by promoting abdominal obesity and/or altering the anatomical structure of the upper respiratory tract (9, 10). Conversely, OSA can also exacerbate hyperandrogenemia in women with PCOS, either directly or indirectly, by inducing INS resistance and reducing levels of sex hormone-binding globulin. PCOS may precede the onset of OSA and contribute to its development and progression; conversely, OSA may also influence the clinical manifestations of PCOS and even aggravate its severity, although the precise mechanisms remain unclear.

Therefore, we designed a two-part study involving a cross-sectional investigation to identify the clinical characteristics and reproductive endocrine and glycolipid metabolic features in women with PCOS and OSA. We also conducted a randomized controlled trial to assess whether CPAP therapy improves reproductive endocrine function and glucose–lipid metabolism and potentially enhances fertility in these patients, thereby providing clinical evidence to inform treatment and prognosis.

## 2 Methods

### 2.1 Study design

A total of 312 infertile patients with PCOS who attended the Department of Reproductive Medicine at Heze Municipal Hospital between February 2023 and December 2024 were initially enrolled, with a mean age of 27.61 ± 4.89 years. Infertility is defined as the failure to achieve clinical pregnancy after at least 12 months of regular, unprotected sexual intercourse (11). After applying the inclusion and exclusion criteria, 294 patients met the inclusion criteria. Eight patients were excluded due to incomplete polysomnography (PSG), six due to the use of ovulation-inducing or lipid-lowering medications, three due to ovarian insufficiency, and one due to severe endocrine diseases.

The study was conducted in two stages. Initially, based on the AHI, a cross-sectional analysis was conducted to compare clinical characteristics and reproductive endocrine and glycolipid metabolic parameters across different OSA severity groups in women with PCOS. Subsequently, a randomized controlled trial was conducted among 101 patients with PCOS and OSA. SPSS 26.0 (random seed = 12,345) was used to generate the allocation sequence, and participants were assigned in a 1:1 ratio to either the CPAP treatment group or the control group. The randomization list and group assignments were managed by two researchers who were not involved in participant recruitment or intervention delivery. Allocation concealment was ensured by placing the allocation sequence in sequentially numbered opaque envelopes, which were opened only after each participant had completed baseline assessments and provided written informed consent.

**Figure 1:**
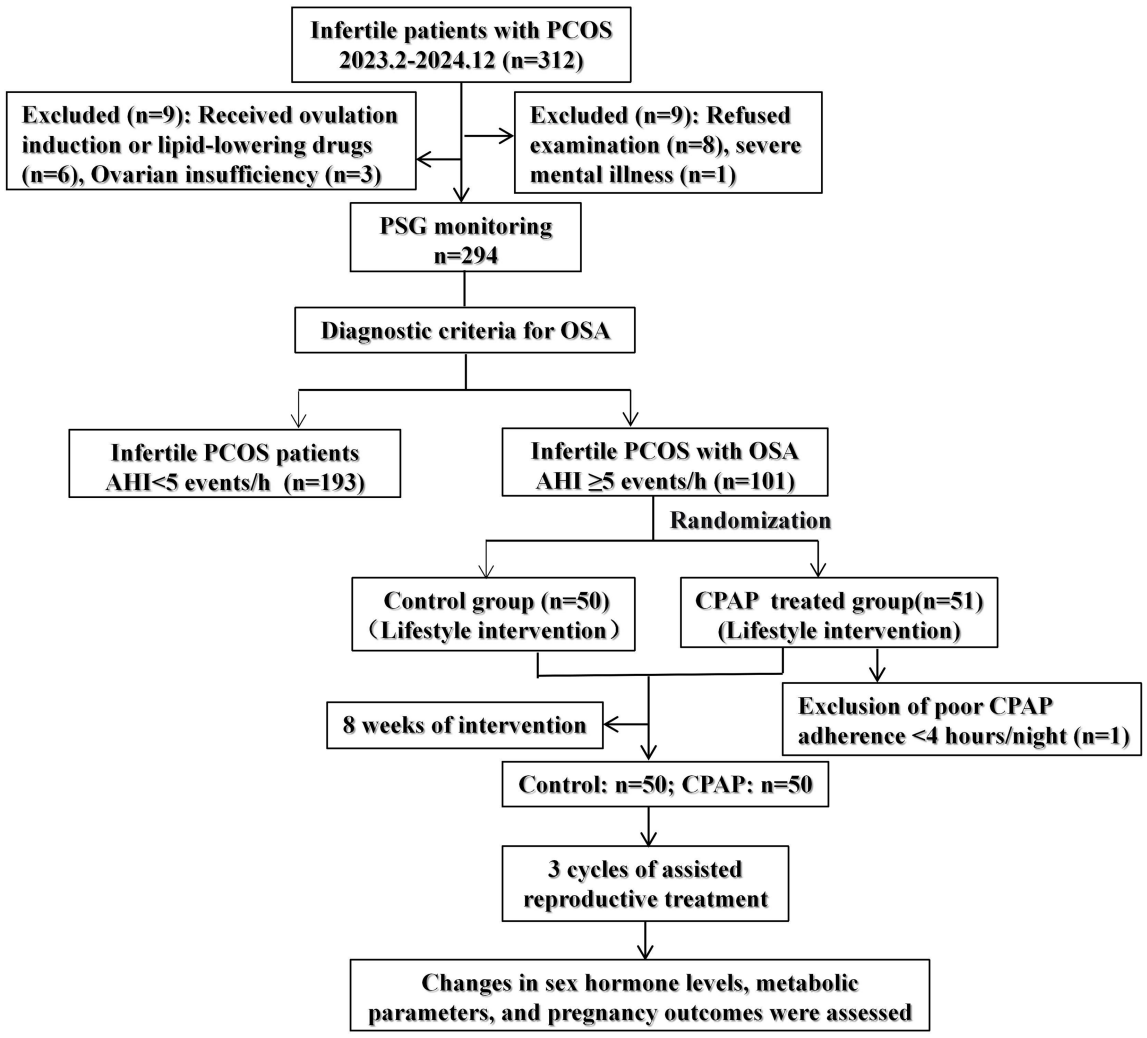
Flow diagram of the study participants. PCOS, polycystic ovary syndrome; AHI, apnea–hypopnea index; PSG, polysomnography; CPAP, continuous positive airway pressure.

The control group received an 8-week lifestyle intervention, including dietary and exercise guidance. The treatment group received CPAP therapy in addition to the same lifestyle intervention for 8 weeks. One participant was excluded due to insufficient CPAP adherence. Following the intervention, both groups underwent three consecutive cycles of assisted reproductive treatment, including ovulation monitoring combined with timed intercourse and artificial insemination by husband (AIH). Additionally, changes in sex hormone levels, metabolic parameters, and pregnancy outcomes were compared between the two groups. Menstrual recovery, ovulation, clinical pregnancy, and miscarriage rates were analyzed for each cycle. A detailed participant flow diagram is shown in Figure 1.

Prior to treatment initiation, the optimal CPAP pressure for each participant was determined through an overnight laboratory titration study (12). Home CPAP adherence was monitored using device-recorded usage data and defined according to established clinical criteria as an average nightly use of ≥4 h per night over the 8-week period (13). Trained research nurses conducted weekly telephone follow-ups to provide clinical support and address potential issues, thereby facilitating adherence. Only one participants was excluded due to insufficient CPAP use. This was attributed to the high treatment adherence among patients seeking infertility treatment, supported by research funding and a comprehensive adherence monitoring system implemented by our team.

This study was conducted in accordance with the Declaration of Helsinki and was approved by the Ethics Committee of Heze Municipal Hospital (Approval No. 2022-KY007). Written informed consent was obtained from all participants prior to enrollment. Participants were clearly informed of the standard treatment for OSA, the randomization design, the possibility of temporary treatment delay, and their right to withdraw at any time and receive standard care immediately. This study was registered in the Chinese Medical Research Registration Information System (Registration No. MR-37-23-031056) as an interventional study.

### 2.2 Patients and eligibility criteria

#### 2.2.1 Inclusion criteria

The inclusion criteria for this study were as follows: (1) age 19–40 years; (2) meeting the diagnostic criteria for PCOS (14): According to the diagnostic criteria for polycystic ovary syndrome formulated by the Chinese Medical Association of Obstetrics and Gynecology in 2018, oligomenorrhea, amenorrhea, or irregular uterine bleeding is a necessary diagnostic condition. Additionally, meeting one of the following two criteria is required for a diagnosis of suspected PCOS: ① clinical manifestations of hyperandrogenism or hyperandrogenemia; ② ultrasound evidence of polycystic changes in the ovaries (≥12 follicles on one side). After meeting the above criteria, other diseases that may cause hyperandrogenemia or ovulatory disorders must be excluded to confirm the diagnosis of PCOS. (3) Diagnostic criteria for OSA (15): Normal (AHI <5 events/h) and OSA (AHI ≥5 events/h). (4) Based on body mass index (BMI), patients were categorized into normal weight (18.5 ≤BMI <24 kg/m^2^), overweight (24 ≤BMI <28 kg/m^2^), and obese BMI ≥ 28 kg/m^2^. All enrolled patients were newly diagnosed with PCOS.

#### 2.2.2 Exclusion criteria

The exclusion criteria for this study are as follows: (1) ovarian insufficiency or endocrine diseases; (2) ovulation induction and hormone treatment within the past 3 months; (3) use of medications affecting glucose–lipid metabolism or antihypertensive drugs within the past 3 months; (4) severe mental illness; and (5) refusal to complete the examinations.

### 2.3 Polysomnography (PSG) protocol

All polysomnographic recordings were performed using a digital sleep monitoring system (model SW-SM2000CB, Compumedics Medical Technology Co., Ltd.) from 22:00 to 06:00 the following morning. A total of seven channels were recorded, including thoracic respiratory effort, abdominal respiratory effort, nasal airflow, snoring, oxygen saturation, pulse rate, and body position. All polysomnographic recordings were scored by two technicians according to the American Academy of Sleep Medicine (AASM) criteria (16, 17). Apnea was defined as a ≥90% reduction in oronasal airflow lasting for ≥10 s. Hypopnea was defined as a 30–90% reduction in airflow lasting for ≥10 s, accompanied by either a ≥3% oxygen desaturation or an arousal. The apnea–hypopnea index (AHI) was calculated as the total number of apnea and hypopnea events per hour of sleep. Based on the AHI, OSA severity was classified as mild (5 ≤AHI <15 events/h), moderate (15 ≤AHI <30 events/h), and severe (AHI ≥30 events/h).

### 2.4 Quantification of hormone concentrations in serum and metabolic parameters

Fasting venous blood samples were collected from all participants between 8:00 and 10:00 a.m. on days 2–5 of the menstrual cycle. In the intervention study, samples were obtained at the same time point before the intervention and after 8 weeks to ensure consistent testing conditions. All samples were centrifuged within 30 min of collection at 3,500 rpm for 10 min at 4 °C. The serum was then separated, aliquoted into cryovials, and stored at −80 °C for batch analysis, avoiding repeated freeze–thaw cycles. The analytical performance of the detection method is presented in Supplementary Data 1.

#### 2.4.1 Reproductive endocrine hormones

Serum levels of anti-Müllerian hormone (AMH), prolactin (PRL), follicle-stimulating hormone (FSH), luteinizing hormone (LH), estradiol (E2), testosterone (T), progesterone (P), and thyroid-stimulating hormone (TSH) were measured using chemiluminescent immunoassays on an automated analyzer (Mindray CL-6000i, Shenzhen, China) with commercial kits according to the manufacturer’s instructions.

#### 2.4.2 Glucose and lipid metabolic parameters

All participants underwent a standard 75-g oral glucose tolerance test (OGTT) after an overnight fast. The glucose solution (75 g dissolved in 300 mL of water) was ingested within 5 min. Venous blood samples were collected at 0 (fasting), 60, and 120 min for the measurement of plasma glucose and insulin levels. OGTT, insulin (INS), total cholesterol (TC), and triglycerides (TGs) were measured using an automated biochemical analyzer (Roche Diagnostics GmbH cobas 8000, Shanghai, China).

### 2.5 Lifestyle intervention

The interventions were administered for 8 weeks. Both groups received a lifestyle intervention. The lifestyle intervention was guided by the evidence-based guidelines for the management of PCOS (18) and was defined as dietary and physical activity modifications aimed at achieving weight loss or preventing weight gain. Daily energy intake was reduced by approximately one-third of the original level, with a total intake of 1,200–1,500 kcal per day, while maintaining a balanced diet. To prevent weight gain and promote overall health, participants were advised to engage in at least 150 min of moderate-intensity physical activity per week, exercise on two non-consecutive days per week incorporating muscle-strengthening activities, or an equivalent combination of both.

### 2.6 Statistical analysis

Statistical analyses were performed using SPSS 26.0 software (IBM Corporation, Armonk, NY, United States). Normally distributed data are presented as mean ± standard deviation (mean ± SD), and non-normally distributed data are expressed as median (25th–75th percentile). For the cross-sectional analysis, a one-way analysis of variance (ANOVA) was used to compare continuous variables across the groups according to the OSA severity. Categorical variables were compared using the chi-squared test. Given the potential confounding effect of obesity, body mass index (BMI) was included as a key covariate in all relevant analyses. Analysis of covariance (ANCOVA) was performed to compare reproductive endocrine and glucose–lipid metabolic parameters between the groups after adjustment for BMI. A multivariable logistic regression analysis was conducted to identify factors independently associated with the presence of OSA in women with PCOS. Clinically relevant variables, including age and BMI, were retained in the model regardless of statistical significance. Other candidate variables were selected based on clinical relevance and univariate analysis. Multicollinearity was assessed using variance inflation factors (VIFs), with VIF of <3 indicating no significant collinearity. The results are presented as odds ratios (ORs) with 95% confidence intervals (CIs).

For the interventional study, comparisons between the groups after the 8-week intervention were performed using independent-samples *t*-tests or the Mann–Whitney *U*-test. The receiver operating characteristic (ROC) curves were constructed to evaluate the predictive value of potential risk factors. The optimal cutoff value was determined using the Youden index, and the area under the curve (AUC), 95% confidence interval (95% CI), sensitivity, and specificity were calculated. A two-sided *p*-value of <0.05 was considered statistically significant. The primary analysis of the intervention study was conducted on a per-protocol (PP) basis, including participants who completed the study. In addition, intention-to-treat (ITT) analysis was performed as a sensitivity analysis to assess the robustness of the findings.

## 3 Results

### 3.1 The incidence of PCOS combined with OSA

A total of 294 patients with PCOS-related infertility were enrolled, with ages ranging from 18 to 40 years. The PSG results showed that the prevalence of OSA was 34.35% (101/294), with OSA patients including 69 cases of mild OSA (23.47%), 23 cases of moderate OSA (7.82%), and 9 cases of severe OSA (3.06%) (Table 1).

**Table 1:** Incidence of PCOS combined with OSA classification.

OSA severity	AHI (events/h)	*N*	Proportion (%)
Non-OSA	<5	193	65.64
Mild OSA	5 ~ 15	69	23.47
Moderate OSA	15 ~ 30	23	7.82
Severe OSA	≥30	9	3.06
Total		294	100

OSA, obstructive sleep apnea; PCOS, polycystic ovary syndrome; AHI, apnea–hypopnea index.

### 3.2 The relationship between baseline characteristics, reproductive endocrine, and glucose–lipid metabolism across different grades of OSA in PCOS patients

PCOS patients with severe OSA had a significantly higher BMI than those without OSA and those with mild or moderate OSA (*F* = 62.931, *p* < 0.001). A univariate ANOVA showed significant differences in serum AMH, T, fasting plasma glucose (FPG), INS, and TG among groups (all *p* < 0.05). After adjusting for BMI using analysis of covariance (ANCOVA), the intergroup difference in FPG was no longer statistically significant (*p* > 0.05). Specifically, the serum AMH level was significantly lower in patients with OSA than in those without OSA (*F* = 8.618, *p* < 0.001), whereas serum T levels were higher (*F* = 2.868, *p* = 0.037). Regarding glucose–lipid metabolic parameters, patients with OSA showed higher fasting insulin (*F* = 6.482, *p* < 0.001) and TG levels (*F* = 5.443, *p* = 0.001) than those without OSA. No significant differences were observed in the other variables after adjusting for BMI (Table 2).

**Table 2:** Baseline characteristics and reproductive endocrine and metabolic indexes by OSA severity in PCOS patients.

Characteristics	Without OSA (*n* = 193)	Mild OSA (*n* = 69)	Moderate OSA (*n* = 23)	Severe OSA (*n* = 9)	*F*/*χ*^2^	*p*
Age (years)	27.36 ± 4.36	27.84 ± 4.94	28.61 ± 5.83	28.78 ± 4.91	0.595	0.619
BMI (kg/m^2^)	23.15 ± 2.98	27.34 ± 3.94^a^	28.25 ± 2.92^a^	34.93 ± 3.79^abc^	62.931	<0.001
Infertility type *n* (%)					2.854	0.460
Primary	148 (76.68%)	48 (69.57%)	15 (65.22%)	6 (66.67%)		
Secondary	45 (23.32%)	21 (30.43%)	8 (34.78%)	3 (33.33%)		
Menstruation *n* (%)					0.811	0.847
Regular	38 (19.69%)	12 (17.39%)	3 (13.04%)	1 (11.11%)		
Irregular	155 (80.31%)	57 (82.61%)	20 (86.96%)	8 (88.89%)		
TSH (μIU/mL)	3.03 ± 1.91	2.74 ± 1.16	3.41 ± 2.10	3.85 ± 4.22	1.808	0.146
E2 (pg/mL)	46.32 ± 17.04	45.63 ± 17.34	45.40 ± 17.31	47.71 ± 14.68	0.350	0.789
P (ng/mL)	0.48 ± 0.25	0.55 ± 0.16	0.47 ± 0.21	0.43 ± 0.10	0.759	0.519
FSH (IU/L)	7.74 ± 2.00	7.21 ± 1.80	7.21 ± 1.54	7.62 ± 0.75	1.558	0.200
LH (IU/L)	8.64 ± 3.74	12.02 ± 5.63	12.11 ± 6.63	12.42 ± 7.94	0.868	0.458
PRL (ng/mL)	22.58 ± 10.69	23.53 ± 13.59	26.12 ± 11.69	28.97 ± 23.49	2.129	0.097
T (ng/mL)	0.53 ± 0.03	0.60 ± 0.04^a^	0.72 ± 0.06^a^	0.79 ± 0.08	2.868	<0.05
AMH (ng/mL)	5.32 ± 0.14	4.17 ± 0.02 ^a^	3.83 ± 0.31^a^	3.74 ± 0.44^a^	8.618	<0.001
FPG (mmol/L)	5.02 ± 0.59	5.18 ± 0.84	5.24 ± 0.13	5.41 ± 0.18	1.453	0.228
OGTT 60 min (mmol/L)	8.25 ± 1.14	8.56 ± 0.84	9.07 ± 1.43	9.56 ± 1.25	2.692	0.067
OGTT 120 min (mmol/L)	6.99 ± 0.79	7.23 ± 2.21	7.86 ± 1.75	8.13 ± 2.52	1.831	0.094
INS (mIU/L)	20.71 ± 1.01	28.06 ± 1.44^a^	29.82 ± 2.26^a^	30.71 ± 3.20^a^	6.482	<0.001
INS 60 min (mIU/L)	169.46 ± 15.98	197.25 ± 46.52	199.78 ± 49.36	203.27 ± 52.65	1.851	0.057
INS 120 min (mIU/L)	135.23 ± 26.48	141.12 ± 42.31	142.12 ± 36.53	143.63 ± 37.23	0.856	0.102
T-CHO (mmol/L)	4.67 ± 1.37	4.73 ± 1.26	4.87 ± 0.93	5.01 ± 1.23	0.536	0.275
TG (mmol/L)	1.72 ± 0.41	1.94 ± 0.06^a^	2.01 ± 0.09^a^	2.25 ± 0.13^abc^	5.443	<0.001

OSA, obstructive sleep apnea; PCOS, polycystic ovary syndrome; BMI, body mass index; TSH, thyroid-stimulating hormone; E2, estradiol; P, progesterone; FSH, follicle-stimulating hormone; LH, luteinizing hormone; PRL, prolactin; T, testosterone; AMH, anti-Müllerian hormone; FPG, fasting plasma glucose; OGTT, oral glucose tolerance test; INS, insulin; T-CHO, total cholesterol; TG, triglyceride.

^a^*p* < 0.05 vs. without OSA, ^b^*p* < 0.05 vs. with mild OSA, ^c^*p* < 0.05 vs. with moderate OSA.

Age and reproductive endocrine and metabolic parameters were compared using ANCOVA adjusted for BMI. Infertility type and menstrual status were not adjusted for BMI.

### 3.3 Logistic regression analysis of the correlation between hormone levels and glucose–lipid metabolic indicators in OSA and PCOS patients

Before using the multiple regression analysis, collinearity diagnostics were performed, and no multicollinearity was observed among the included variables (VIF <3 for all variables). A logistic regression analysis was performed with the PCOS-OSA group as the dependent variable and variables showing statistical significance in the univariate analysis as independent variables. BMI was included as a covariate to account for the potential confounding effect of obesity. Age, BMI, FSH, T, FPG, INS, and TG were all treated as continuous variables, as shown in Table 3. Our results analysis indicated that higher BMI [odds ratio (OR): 1.379, 95% CI: 1.261–1.567], INS (OR: 1.052, 95% CI: 1.027–1.108), and TG levels (OR 2.276, 95% CI: 1.276–4.965) were independently associated with an increased risk of OSA in patients with PCOS (*p* < 0.05). Conversely, higher AMH levels were associated with a reduced risk of OSA (OR 0.71, 95% CI: 0.562–0.915, *p* < 0.05). These results indicate that, even after adjusting for BMI, hyperinsulinemia, hypertriglyceridemia, and low AMH levels remain independently associated with OSA in patients with PCOS.

**Table 3:** Logistic regression analysis on OSA occurrence in PCOS patients.

Variables	*B*	SE	Wald	*p*	Exp (*B*)	95 (%) CI lower–upper
Age (years)	−0.010	0.034	0.084	0.772	1.003	0.924–1.008
BMI (kg/m^2^)	0.347	0.055	37.710	<0.001	1.379	1.261–1.567
FSH (IU/L)	−0.104	0.106	0.971	0.324	0.902	0.733–1.109
T (ng/mL)	0.880	0.598	2.187	0.139	2.411	0.747–7.779
AMH (ng/mL)	−0.332	0.124	7.356	<0.001	0.710	0.562–0.915
FPG (mmol/L)	0.347	0.322	1.188	0.276	0.707	0.376–1.330
INS (mIU/L)	0.065	0.019	11.297	<0.001	1.052	1.027–1.108
TG (mmol/L)	0.923	0.347	7.107	<0.001	2.276	1.276–4.965

BMI, body mass index; FSH, follicle-stimulating hormone; T, testosterone; AMH, anti-Müllerian hormone; FPG, fasting plasma glucose; INS, insulin; TG, triglyceride; CI, confidence interval.

Logistic regression analysis (enter method) was performed with BMI as the first entered variable. No multicollinearity was detected (VIF <3).

### 3.4 ROC curve analysis of risk factor-based prediction model for OSA in women with PCOS

**Figure 2:**
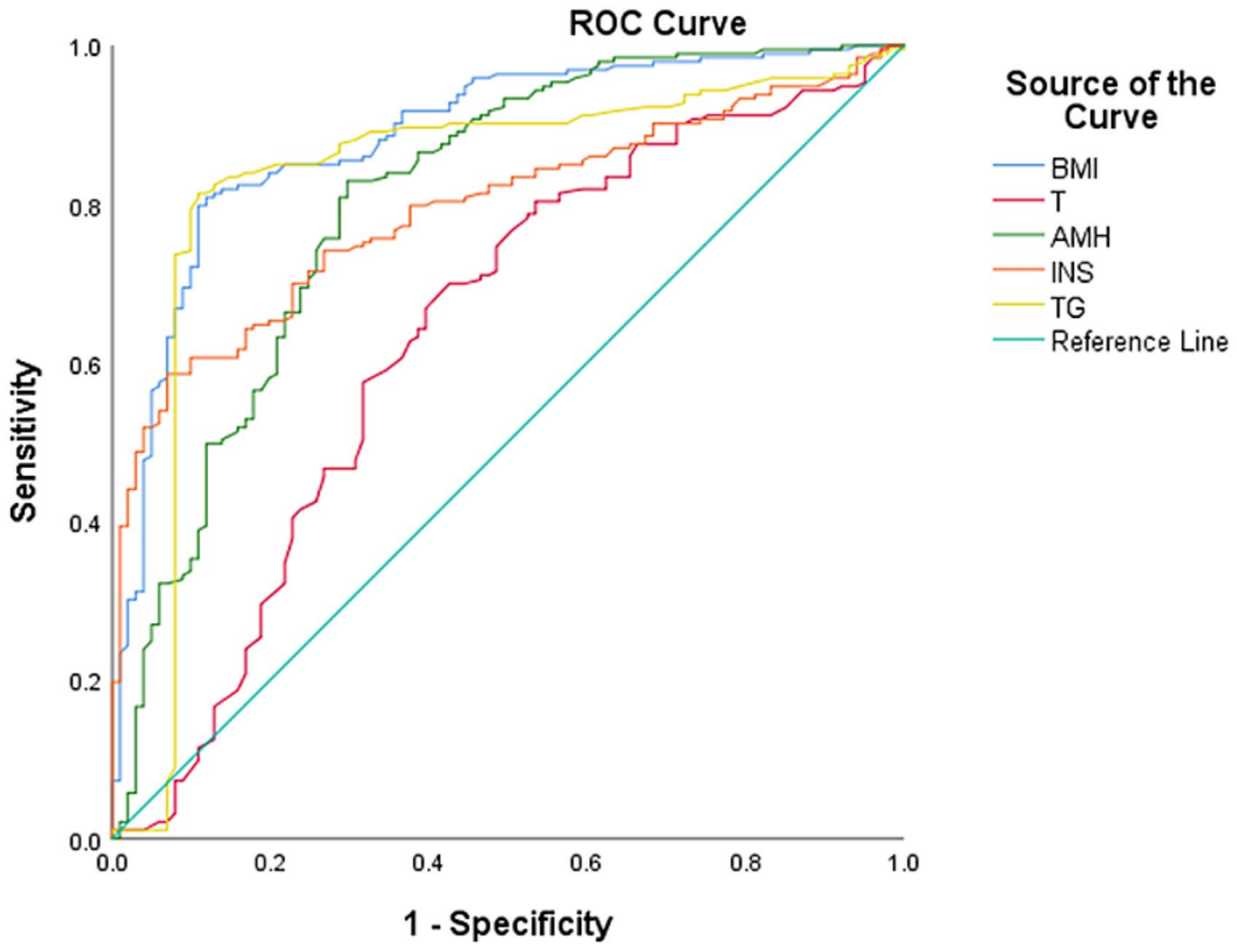
Receiver operating characteristic of risk factors for PCOS with OSA. 1-specificity (*x*-axis, representing the false positive rate) is defined as the horizontal axis, and sensitivity (*y*-axis, representing the true positive rate) is defined as the vertical axis. The receiver operating characteristic (ROC) curves of five risk factors, namely the body mass index (BMI), testosterone (T), anti-Müllerian hormone (AMH), insulin (INS), and triglycerides (TG), are plotted. The diagonal line in the figure denotes the reference line for no predictive value, corresponding to an area under the curve (AUC) of 0.5.

The ROC curve parameters of each indicator in the risk factor prediction model for OSA in women with PCOS are summarized in Table 4, including optimal cutoff values, AUC, *p*-values, 95% confidence intervals (95% CI), sensitivity, and specificity. All indicators showed statistically significant discriminatory ability (*p* < 0.001) (Figure 2). BMI showed an AUC of 0.885 (95% CI: 0.845–0.926), with an optimal cutoff value of 24.05 kg/m^2^ (sensitivity: 80.8% and specificity: 88.1%). Triglycerides (TG) showed an AUC of 0.834 (95% CI: 0.777–0.891), with an optimal cutoff value of 1.77 mmol/L (sensitivity: 89.1% and specificity: 80.8%). Insulin (INS) showed an AUC of 0.794 (95% CI: 0.744–0.844), with an optimal cutoff value of 20.43 mIU/L (sensitivity: 90.1% and specificity: 60.6%). AMH showed an AUC of 0.806 (95% CI: 0.750–0.862), with an optimal cutoff value of 4.0 ng/mL (sensitivity: 81.9% and specificity: 70.3%). Testosterone (T) showed an AUC of 0.637 (95% CI: 0.567–0.707), with an optimal cutoff value of 0.59 ng/mL (sensitivity: 66.8% and specificity: 60.4%).

**Table 4:** ROC curve analysis of the predictive model for PCOS and OSA.

Variable	Cutoff	AUC	*p*	95% CI lower–upper	Sensitivity (%)	Specificity (%)
BMI (kg/m^2^)	24.05	0.885	<0.001	0.845–0.926	80.8	88.1
T (ng/mL)	0.59	0.637	<0.001	0.567–0.707	66.8	60.4
AMH (ng/mL)	4	0.806	<0.05	0.750–0.862	81.9	70.3
INS (mIU/L)	20.43	0.794	<0.001	0.744–0.844	90.1	60.6
TG (mmol/L)	1.77	0.834	<0.05	0.777–0.891	89.1	80.8

ROC, receiver operating characteristic; PCOS, polycystic ovary syndrome; BMI, body mass index; T, testosterone; AMH, anti-Müllerian hormone; INS, insulin; TG, triglyceride.

### 3.5 Comparison of general baseline data between the two groups

Baseline clinical characteristics (age, BMI, infertility type, and menstrual status) and laboratory parameters (reproductive endocrine and glucose–lipid metabolic indicators) were compared between the two groups. No significant differences were observed in any baseline clinical or laboratory variables between the control and CPAP treatment groups (*p* > 0.05), indicating good baseline comparability prior to treatment initiation (Table 5).

**Table 5:** Comparison of general baseline characteristics between the control group and the CPAP treatment group.

Characteristics	Control (*n* = 50)	CPAP treatment (*n* = 51)	*T*/*χ^2^*	*p*
Age (years)	27.00 ± 4.10	26.18 ± 3.85	1.064	0.303
BMI (kg/m^2^)	24.09 ± 4.47	25.29 ± 3.34	0.447	0.252
Infertility type *n* (%)				
Primary	37 (74.00%)	31 (60.08%)	2.005	0.157
Secondary	13 (26.00%)	20 (39.20%)		
Menstruation *n* (%)				
Regular	14 (28.00%)	15 (29.40%)	0.025	0.857
Irregular	36 (72.00%)	36 (70.60%)		
TSH (μIU/mL)	3.20 ± 2.35	3.05 ± 1.44	0.945	0.704
E2 (pg/mL)	41.36 ± 14.53	46.81 ± 13.39	0.281	0.053
P (ng/mL)	0.44 ± 0.23	0.52 ± 0.27	0.168	0.683
FSH (IU/L)	7.83 ± 1.99	7.62 ± 1.68	0.023	0.564
LH (IU/L)	11.85 ± 5.14	12.71 ± 5.46	0.099	0.557
PRL (ng/mL)	22.82 ± 10.37	28.47 ± 13.81	0.543	0.055
T (ng/mL)	0.60 ± 0.28	0.64 ± 0.31	0.603	0.514
AMH (ng/mL)	4.67 ± 1.83	4.47 ± 1.69	0.269	0.573
FPG (mmol/L)	5.16 ± 0.99	5.10 ± 0.56	0.209	0.702
INS (mIU/L)	25.41 ± 3.98	25.79 ± 3.70	0.332	0.836
TG (mmol/L)	2.16 ± 0.31	2.22 ± 0.41	0.282	0.375

OSA, obstructive sleep apnea; PCOS, polycystic ovary syndrome; BMI, body mass index; AMH, anti-Müllerian hormone; FSH, follicle-stimulating hormone; LH, luteinizing hormone; E2, estradiol; T, testosterone; A, androstenedione; PRL, prolactin; TSH, thyroid-stimulating hormone; FPG, fasting plasma glucose; OGTT, oral glucose tolerance test; INS, insulin; T-CHO, total cholesterol; TG, triglyceride.

### 3.6 Changes in reproductive hormones and glucose–lipid metabolism in the CPAP treatment and the control groups

In the CPAP treatment group, serum T levels were significantly lower than those in the control group (*t* = 3.471, *p* = 0.019). Furthermore, the CPAP treatment group exhibited significantly reduced levels of FPG (*t* = 3.420, *p* = 0.025), INS (*t* = 4.175, *p* < 0.001), and TG (*t* = 4.680, *p* < 0.001) compared with the control group. No significant differences were observed between the groups in terms of TSH, FSH, E2, P, LH, PRL, and AMH levels (*p* > 0.05) (Table 6).

**Table 6:** Changes in reproductive hormone and glucose–lipid metabolic parameters in the CPAP treatment group and the control group.

Parameter	Control (*n* = 50)	CPAP treated (*n* = 50)	*t*	*p*
TSH (μIU/mL)	2.70 ± 1.42	3.91 ± 4.49	2.187	0.071
FSH (IU/L)	7.95 ± 1.81	7.24 ± 1.94	1.353	0.061
LH (IU/L)	13.18 ± 7.51	10.61 ± 5.85	1.573	0.058
E2 (pg/mL)	47.58 ± 13.38	44.90 ± 15.45	0.184	0.354
P (ng/mL)	0.48 ± 0.21	0.58 ± 0.73	1.517	0.343
T (ng/mL)	0.69 ± 0.28	0.54 ± 0.33	3.471	0.019
PRL (ng/mL)	21.85 ± 8.57	22.64 ± 11.99	1.956	0.705
AMH (ng/mL)	4.62 ± 1.73	3.99 ± 2.19	1.373	0.054
FPG (mmol/L)	5.28 ± 0.84	4.94 ± 0.66	3.420	<0.05
INS (mIU/L)	30.83 ± 10.91	20.54 ± 11.36	4.175	<0.001
TG (mmol/L)	2.23 ± 0.58	1.73 ± 0.53	4.680	<0.001

CPAP, continuous positive airway pressure; OSA, obstructive sleep apnea; PCOS, polycystic ovary syndrome; BMI, body mass index; AMH, anti-Müllerian hormone; FSH, follicle-stimulating hormone; LH, luteinizing hormone; E2, estradiol; T, testosterone; A, androstenedione; PRL, prolactin; TSH, thyroid-stimulating hormone; FPG, fasting plasma glucose; OGTT, oral glucose tolerance test; INS, insulin; T-CHO, total cholesterol; TG, triglyceride.

### 3.7 Comparison of menstrual recovery, ovulation, and pregnancy outcomes between the CPAP treatment group and the control group

The clinical pregnancy rate was significantly higher in the CPAP treatment group than in the control group (38.0% vs. 19.6%, *χ*^2^ = 4.173, *p* = 0.041). Although the CPAP treatment group also showed a higher menstrual recovery rate (58.0% vs. 43.1%), a higher ovulation rate (54.0% vs. 35.3%), and a lower miscarriage rate (26.3% vs. 40.0%) compared with the control group, these differences were not significant (all *p* > 0.05) (Table 7).

**Table 7:** Comparison of menstrual recovery, ovulation, and pregnancy outcomes of the study subjects before and after treatment [*n* (%)].

Group	Menstruation [*n* (%)]	Ovulation [*n* (%)]	Pregnancy rate [*n* (%)]	Miscarriage rate [*n* (%)]
Control group (*n* = 50)	22 (43.1%)	18 (35.3%)	10 (19.6%)	4 (40.0%)
CPAP-treated group (*n* = 50)	29 (58.0%)	27 (54.0%)	19 (38.0%)	5 (26.3%)
*χ* ^2^	2.231	3.576	4.173	0.573
*p*	0.135	0.059	<0.050	0.449

CPAP, continuous positive airway pressure.

## 4 Discussion

Women with PCOS have a 30-fold higher risk of developing OSA compared with those without PCOS (19). Although obesity is prevalent among women with PCOS and OSA, the severity of OSA (as measured by AHI) cannot be solely attributed to an increased BMI (20). Recent studies have focused on the relationship between OSA and PCOS (21), with reproductive doctors reporting that sleep-disordered breathing is rarely addressed in the management of PCOS. The reported prevalence of OSA in women ranges from 9 to 28%, whereas women with PCOS exhibit a significantly higher incidence (5). This study examined the reproductive endocrine and glucose–lipid metabolic characteristics associated with OSA in women with PCOS and further investigated whether improving sleep quality through CPAP therapy can indirectly improve endocrine, metabolic, and reproductive outcomes in infertile women with PCOS.

Obesity is a well-recognized risk factor for PCOS, OSA, and metabolic dysfunction (22, 23). More than half of women with PCOS are overweight or obese (24), and excess adipose tissue contributes to upper airway narrowing and impaired ventilatory control, thereby directly promoting the development of OSA (25). Although BMI is a major shared risk factor for both PCOS and OSA, our findings suggest that the observed associations are not entirely attributable to obesity. After adjusting for BMI as a continuous covariate in ANCOVA, the PCOS with OSA group continued to exhibit significantly higher T, fasting INS, and TG levels, and lower AMH levels; however, residual confounding cannot be entirely excluded. Logistic regression models further adjusted for age and BMI, low AMH, high INS, and high TG levels remained independent risk factors for patients with PCOS and OSA, suggesting that they may be associated with reproductive endocrine abnormalities through mechanisms independent of obesity. These results support the notion that OSA exerts independent effects on endocrine and metabolic dysfunction.

A logistic regression analysis indicated that BMI had the highest OR among the identified factors and was an independent risk factor for OSA in patients with PCOS (OR: 1.379), which is consistent with previous studies (22). Excess adipose tissue contributes to upper airway narrowing, impaired ventilatory control, and increased chest wall resistance, representing direct pathogenic mechanisms underlying OSA (26). Intermittent hypoxia associated with OSA may trigger oxidative stress and inflammatory responses, leading to adipose tissue dysfunction and dysregulated lipid metabolism, which may result in hypertriglyceridemia and the development or exacerbation of insulin resistance (27). Patients with PCOS frequently exhibit insulin resistance and metabolic abnormalities, which may be further aggravated by OSA-related intermittent hypoxia (28). Hyperinsulinemia can stimulate ovarian androgen production, disrupt the reproductive axis, and impair ovulation (29). In turn, hyperandrogenism may increase respiratory instability and lower the apnea threshold (30), thereby predisposing women with PCOS to OSA. Sleep-disordered breathing may also disrupt the hypothalamic regulation of gonadotropin-releasing hormone secretion, alter LH and FSH pulsatility (31), and further exacerbate endocrine and menstrual dysfunction in PCOS.

Our study showed that serum AMH levels were significantly lower in PCOS women with OSA, and OSA was independently associated with lower AMH levels even after adjusting for BMI. OSA may affect granulosa cell function through pathways such as intermittent hypoxia, oxidative stress, and insulin resistance (32). Hyperandrogenism can disrupt the follicular microenvironment and inhibit granulosa cell function, further affecting ovarian reserve (33). However, the present study cannot determine whether low AMH is a consequence of OSA or whether both conditions arise from shared metabolic and endocrine disturbances. Using an ROC model to predict OSA risk in infertile patients with PCOS, a BMI of ≥24.05 kg/m^2^ was associated with an increased risk of OSA, slightly higher than the conventional overweight threshold (24 kg/m^2^) and the clinical risk cutoff for PCOS women. This finding suggests that early-stage overweight patients with PCOS should be prioritized for PSG screening to allow timely identification and intervention for high-risk individuals. These results support the proposed mechanistic relationships.

Research indicates that (34) sleep disturbances are associated with reduced ovarian reserve, potentially due to intermittent hypoxia caused by OSA in patients with PCOS. This hypoxia may exacerbate insulin resistance, worsen endocrine disorders, stimulate ovarian androgen secretion, disrupt the follicular microenvironment, and inhibit granulosa cell function, ultimately impairing ovarian reserve. In our study, FSH levels in the CPAP-treated group showed a decreasing trend (7.95 ± 1.81 vs. 7.24 ± 1.94 IU/L), and LH levels also tended to decrease following CPAP therapy (13.18 ± 7.51 vs. 10.61 ± 5.85 IU/L). Although these changes were not significant, they suggest that CPAP may partially restore normal sleep architecture and indirectly modulate GnRH pulsatility, thereby attenuating LH predominance in patients with PCOS.

CPAP is the recommended first-line treatment for OSA (35), as it can alleviate related symptoms and improve metabolic disturbances. However, evidence regarding its effects in patients with PCOS, particularly on reproductive potential and endocrine homeostasis, remains limited (36). We selected an 8-week CPAP intervention duration based on previous well-validated clinical studies on PCOS and OSA. Tasali et al. (37) demonstrated that 8 weeks of CPAP significantly improved insulin sensitivity and reduced the AHI in obese women with PCOS and OSA. Consistent with these findings, significant reductions in fasting insulin (*p* < 0.001) and serum T (*p* = 0.019) levels were observed following CPAP treatment, supporting the hypothesis that improved sleep quality and nocturnal oxygenation may indirectly restore endocrine homeostasis in patients with PCOS and OSA. Previous studies have emphasized that these metabolic and hormonal benefits are largely independent of weight change, highlighting the direct role of sleep-related mechanisms beyond obesity. Improved sleep quality may have a beneficial effect on the clinical manifestations of this syndrome (38).

The results of this study demonstrated that, after 8 weeks of CPAP therapy followed by three cycles of ovulation induction and AIH, pregnancy outcomes were significantly improved. The clinical pregnancy rate in the CPAP treatment group was significantly higher than that in the control group (38.0% vs. 19.6%, *p* = 0.041). Although the miscarriage rate was lower in the CPAP treatment group than in the control group (26.3% vs. 40.0%), this difference was not statistically significant (*p* = 0.449). These findings suggest that CPAP therapy may improve pregnancy outcomes in PCOS infertile women with OSA primarily through metabolic and endocrine improvements in combination with assisted reproductive treatment rather than the independent effect of CPAP therapy.

This study has some limitations. First, the absence of a healthy non-PCOS control group limited the ability to assess PCOS as an independent risk factor for OSA. Furthermore, the moderate sample size and small CPAP intervention cohort (50 vs. 50) reduced the statistical power of the pregnancy outcome analyses and precluded subgroup assessments based on OSA severity or BMI. Second, the 8-week CPAP intervention demonstrated only short-term benefits for metabolism and clinical pregnancy; long-term effects on endocrine regulation and reproductive outcomes, such as live birth and pregnancy complications, remain unclear and require extended follow-up. Third, adjustment for BMI could not fully eliminate obesity as a confounding factor. Given its shared role in PCOS and OSA and its association with insulin resistance and hyperandrogenism, the independent effects of OSA on reproductive and metabolic outcomes cannot be isolated. Finally, the combination of CPAP therapy with ovulation induction and assisted reproductive treatments may have confounded the observed effects on fertility outcomes, making it difficult to isolate the independent contribution of CPAP.

## 5 Conclusion

Overall, OSA is common in women with PCOS and is associated with adverse metabolic and endocrine profiles. CPAP therapy may contribute to improvements in metabolic parameters and potentially enhance reproductive outcomes when combined with standard fertility treatments. Further large-scale and long-term studies are needed to confirm these findings.

## Data Availability

The original contributions presented in the study are included in the article/Supplementary material, further inquiries can be directed to the corresponding authors.
