# Supplementary material for: Clinical characteristics and pregnancy outcomes in polycystic ovary syndrome complicated by obstructive sleep apnea: a correlative study
Source: Front Med (Lausanne). 2026 Apr 15;13:1714294. doi: 10.3389/fmed.2026.1714294 (PMC13125096; doi:10.3389/fmed.2026.1714294)
Supplement: Supplementary file 1 [file Table_1.docx]

|  | sensitivity | detection range | intra-assay CV | inter-assay CV |
| --- | --- | --- | --- | --- |
| FSH | 0.2 mIU/mL | 1.0mIU/mL-200mIU/mL | ≤3.0% | ≤5.0% |
| LH | 0.2 mIU/mL | 0.4mIU/mL-100mIU/mL | ≤3.0% | ≤5.0% |
| PRL | 0.47ng/mL | 0.47 ng/mL-200ng/mL | ≤3.0% | ≤5.0% |
| T | 0.1 ng/mL | 0.1 ng/mL-16.0ng/mL | ≤3.0% | ≤5.0% |
| P | 0.1 ng/mL | 0.2ng/mL-40ng/mL | ≤3.0% | ≤5.0% |
| E2 | 5 pg/mL | 40pg/mL-1000pg/mL | ≤3.0% | ≤5.0% |
| TSH | 0.01µIU/mL | 0.01μIU/mL-100μIU/mL | ≤3.0% | ≤5.0% |
| INS | 0.2 μIU/mL | 0.2μIU/mL-1000μIU/mL | ≤3.0% | ≤5.0% |
| FPG | 0.11 mmol/L | 0.11mmol/L-41.6mmol/L | ≤2.0% | ≤3.0% |
| TG | 0.1 mmol/L | 0.1mmol/L-10mmol/L | ≤2.0% | ≤3.0% |
| TC | 0. 1 mmol/L | 0.1mmol/L-20mmol/L | ≤3.0% | ≤4.0% |

Table Supplementary Data 1
